# Litigations in orthopedics and trauma surgery: reasons, dynamics, and profiles

**DOI:** 10.1007/s00402-021-03958-1

**Published:** 2021-05-27

**Authors:** Martin Gathen, M. Jaenisch, F. Fuchs, L. Weinhold, M. Schmid, S. Koob, D. C. Wirtz, M. D. Wimmer

**Affiliations:** 1 Department of Orthopaedics and Trauma Surgery, University Hospital of Bonn, Venusberg-Campus 1, 53127 Bonn, Germany; 2 Institute of Medical Biometry, Informatics and Epidemiology, University Hospital of Bonn, Bonn, Germany

**Keywords:** Litigation, Lawsuit, Orthopedic surgery, Treatment errors, Medical malpractice

## Abstract

**Background:**

In recent literature, the increasing number of medical litigations, both in terms of the number of cases being filed and the substantive costs associated with lawsuits, has been described. This study aims to provide an overview of the profile of litigation for orthopedic and trauma surgery to describe the differences and the development of the number of cases over time.

**Patients and Materials:**

A retrospective review of all litigations between 2000 and 2017 was conducted using the institutional legal database. The causes of litigation were documented and classified into seven major categories. In addition to plaintiff characteristics, the litigation outcomes and the differences between emergency and elective surgery were analyzed.

**Results:**

A total of 230 cases were evaluated. The mean age of the plaintiffs was 44.6 ± 20.1 years, and 56.8% were female. The main reasons for litigation were claimed inappropriate management (46.1%), misdiagnosis (22.6), and poor nursing care (8.3%). Significantly more litigations were filed against surgeons of the orthopedic subspecialty compared with trauma surgeons (78%; *p* ≤ 0.0001). There were significantly fewer litigations per 1000 cases filed overall in 2009–2017 (65% less; *p* = 0.003) than in 2000–2008.

**Conclusion:**

Our results could not confirm the often-stated trend of having more litigations against orthopedic and trauma surgeons. Although the absolute numbers increased, the number of litigations per 1000 patients treated declined. Patients who underwent elective surgery were more likely to file complaints than emergency patients.

## Introduction

The field of orthopedic surgery has a high incidence of medical malpractice claims, resulting in a relative risk of 99% for each surgeon to receive at least one claim in his/her career [1]. Many publications have described an increase in the number of lawsuits against medical professionals across all fields of practice [2–5]. The increasing demands on the patient side potentially influenced by incorrect information communicated through the internet or social media platforms, the escalating willingness to regress and litigate, and the misconception about the limits of the medically possible outcomes are considered potential reasons [6–9]. Patient satisfaction and patient or customer service have been neglected for a long time by the medical community compared with commercial branches [10]. Studies have demonstrated that physicians who received low patient satisfaction ratings were more likely to have malpractice lawsuits than those with high ratings [8]. Training programs for patient-centered communication and empathy and intervention programs for improving patient satisfaction are recent prevention strategies [11–14].

Nevertheless, litigations require extensive resources and produce considerable expenses for society and healthcare systems. In 2000–2006, more than US$ 321 million were paid in orthopedic surgery-related settlements in the United Kingdom [15]. The cost of defending US malpractice claims, including legal costs, awards, and underwriting costs, was estimated at US$ 6.5 billion in 2001 [16, 17].

Only a few studies have examined the subject of litigation in orthopedic and trauma surgery and investigated the main reasons, profiles, and trends. Therefore, this study aims to give an overview of the current status of litigation procedures in orthopedic and trauma surgery by evaluating the cases of a level I trauma center in Germany. The authors further seek to evaluate the claimed increase in litigation procedures in the field of orthopedic and trauma surgery. In addition, the differences between cases of elective and emergency surgery and the changes in the dynamics of the profile of litigation procedures over time are presented.

## Patients and methods

A retrospective single-center cohort study was performed by analyzing all litigations filed between January 2000 and December 2017. The institution is a 120-bed university hospital serving as a tertiary care center for spine surgery and arthroplasty. The institution is also a level I trauma center. The study was approved by the local institutional review board (No. 080/19).

Data were obtained from the legal department’s database. The inclusion criterion was that the cases required legal assistance in the time period described. Cases involving minor complaints that were not dealt with by the legal department were excluded. The cases were anonymized and examined by two investigators. Epidemiological data and plaintiff characteristics, including age, sex, and medical history, were collected (Table 1). The causes for litigation were documented and classified into seven major categories (A–G). Additional subgroups have been identified for a more detailed examination (Tables 2, 3). The stated symptoms and complaints resulting from the potential malpractice were classified into eight major categories (A–H) and further subgroups (Table 4).

**Table 1:** Descriptive statistics and overview of the most important variables

Characteristics		
*N*	230	
Period (≤ 2008), *n* (%)	88	38.3%
Period (> 2008), *n* (%)	142	61.7%
Orthopedic, *n* (%)	157	68.3%
Trauma surgery, *n* (%)	73	31.7%
Age (time of treatment), mean (SD)	47.6	20.1
Sex male (%)	99	43.2%
Time (years) between treatment and litigation, median (range)	1	0–9
Liability, *n* (%)	55	23.9%
Liability exist	121	52.6%
No liability	1	0.4%
Missing data	53	23%
Causes of litigation, main categories, *n* (%)		
A2: inappropriate management/mistreatment/failure to apply fixation	106	46.1%
A1: misdiagnosis/delay/failure to diagnose (frx-dislocation-rupture)	52	22.6%
G2: poor nursing care, inappropriate moving by staff, injuries in hospital, etc	19	8.3%
B1: iatrogenic nerve damage	18	7.8%
Symptoms and complaints. main categories, *n* (%)	290	
H4: mobility problems	108	37.2%
H1: discomfort and pain	46	15.9%
E3: poor surgery outcome requiring reoperation	44	15.2%
B1: Iatrogenic nerve damage	27	9.3%

**Table 2:** List of all categories concerning the grounds for litigation sorted by orthopedic and trauma surgery

Categories for causes of litigation	Absolute		Percentage	
	Orth	Trauma	Orth	Trauma
A1: misdiagnosis/delay/failure to diagnose (frx-dislocation-rupture)	31	21	19.7	28.8
A2: inappropriate management/mistreatment/failure to apply fixation	73	33	46.5	45.2
B1: iatrogenic nerve damage	12	6	7.6	8.2
B2: latrogenic damages (fracture, tendon, or artery rupture)	5	2	3.2	2.7
C1: infections	4	1	2.5	1.4
D4: allergies	1	0	0.6	0
E1: inappropriate metal work placement	5	0	3.2	0
E2: incorrect/inappropriate/poor surgery (no reference for second intervention)	4	3	2.5	4.1
E3: poor surgery outcome requiring reoperation	2	2	1.3	2.7
F1: non-union	1	0	0.6	0
G1: inadequate follow-up	2	1	1.3	1.4
G2: poor nursing care, inappropriate moving by staff, injuries in hospital, etc	15	4	9.6	5.5
G3: no consent	2	0	1.3	0

**Table 3:** List of all categories concerning symptoms and complaints after treatment sorted by orthopedic and trauma surgery

Categories for stated symptoms and complaints	Absolute		Percentage	
	Orth	Trauma	Orth	Trauma
A1: misdiagnosis/delay/failure to diagnose (frx-dislocation-rupture)	1	0	0.5	0
A2: inappropriate management/mistreatment/failure to apply fixation	2	0	1	0
B1: iatrogenic nerve damage	21	6	10.3	7
B2: iatrogenic damages (fracture, tendon, or artery rupture)	5	7	2.5	8.1
C1: infections	14	2	6.9	2.3
C3: skin problems/pressure sores	13	3	6.4	3.5
D3: bleeding	1	0	0.5	0
D4: allergies	2	1	1	1.2
D6: death	5	2	2.5	2.3
E3: poor surgery outcome requiring reoperation	33	11	16.2	12.8
G1: inadequate follow-up	1	1	0.5	1.2
G2: poor nursing care, inappropriate moving by staff, injuries in hospital, etc	2	1	1	1.2
H1: discomfort and pain	31	15	15.2	17.4
H2: amputation	0	1	0	1.2
H3: deformity	0	1	0	1.2
H4: mobility problems	73	35	35.8	40.7

**Table 4:** Target variable number of litigations per 1000 cases

Characteristic	RR	CI (lower)	CI (upper)	Pr ( > |*z*|)
Intercept	1.09	0.79	1.46	0.5969
Second time period (2009–2017)	0.44	0.32	0.61	< 0.0001
Orthopedic	1.74	1.26	2.42	0.0001

The litigation outcomes and differences between emergency and elective surgery were also analyzed. The entire investigation period was divided into two equivalent periods of time (2000–2008 and 2009–2017). These time periods were compared in terms of the rate of complaints. Further, the relative number of litigations in relation to the absolute number of patients treated was analyzed.

### Statistical analysis

The data characteristics were described as the means with standard deviations (SD) for the continuous variables and frequency distributions with percentages for the categorical variables. The response variables were defined as the number of litigations related to the treatment years 2000–2017 and the number of litigations resulting in a favorable outcome for the plaintiff. The differences between trauma surgery and orthopedics and between the periods 2000–2008 and 2009–2017 regarding litigations that resulted in existing liability for the accused were assessed descriptively.

Further, we examined the effects of the explanatory variables *subspecialties* (trauma surgery vs. orthopedics) and *time period* (2000–2009 vs. 2010–2017) on the number of litigations using a negative binomial regression model. Each year was considered one unit of observation. For easy interpretation, we included the variable *number of treatments* (in 1000) as an offset in the regression model. Consequently, the effect estimates could be interpreted at the level of *number of litigations per 1000 treatments*. The estimates obtained from the model were presented as rate ratios (RR) with a 95% confidence interval (CI) for the negative binomial regression model. *p *values < 0.05 were considered significant. All analyses were carried out using the R Software for Statistical Computing version 4.0.3.

## Results

**Fig. 1:**
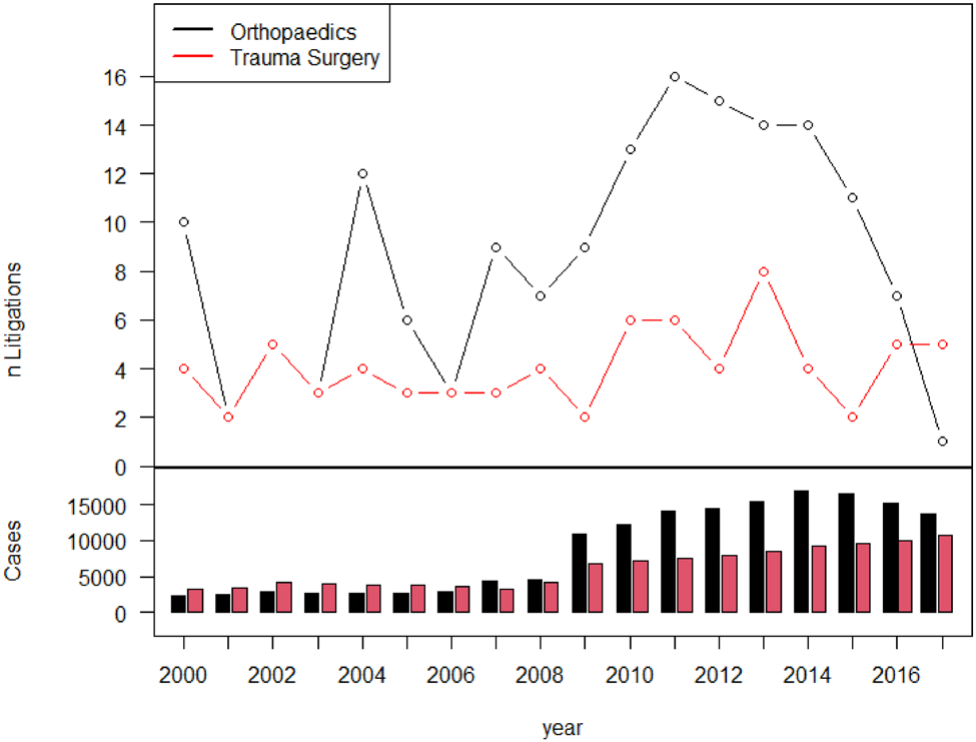
Upper half: absolute number of cases filed per year. Lower half: absolute number of patients treated (inpatient = red/outpatient = black)

**Fig. 2:**
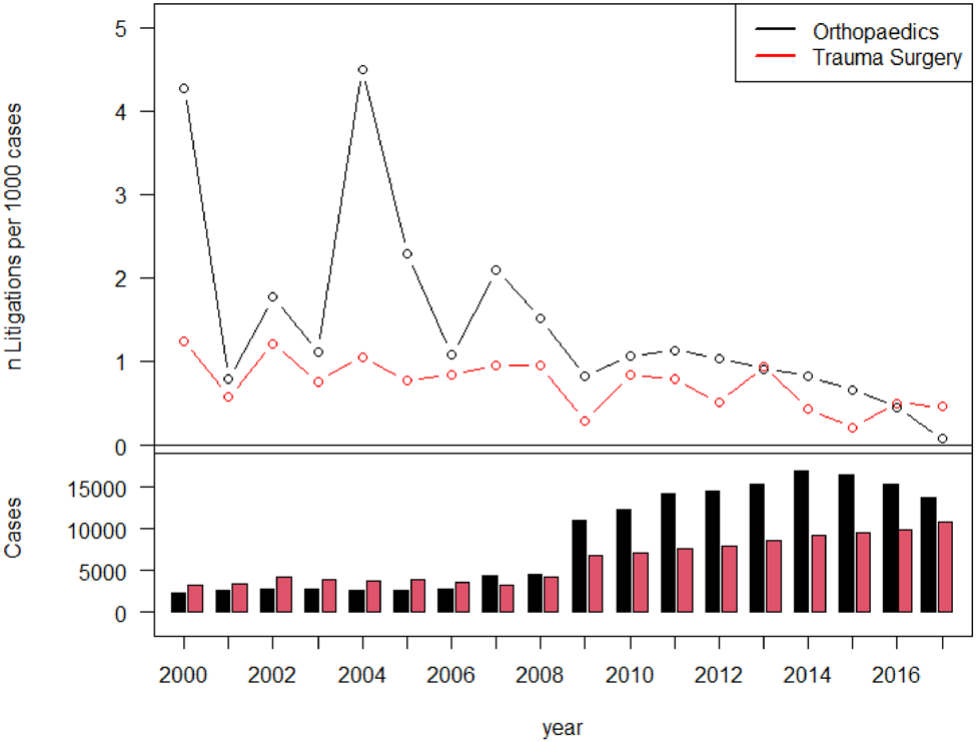
Upper half: rate of lawsuits per 1000 cases per year. Lower half: absolute number of patients treated (inpatient = red/outpatient = black)

For over a period of 18 years (2000–2017), *n* = 267,882 cases were treated and resulted in *n* = 230 cases of litigation (0.086%). The mean age of the plaintiffs was 47.6 ± 20.1 (range 0–99) years. In total, 43.2% of the patients (*n* = 99) were male, and 56.8% (*n* = 131) were female. The number of patients treated increased steadily over the course of the observed period (Fig. 1). In 2000, there were 3810 patients treated in the outpatient area and 1752 inpatient patients. There were 5111 outpatients and 2,365 inpatient cases in 2008 and 20,287 outpatients and 4209 inpatient cases in 2017. The total number of cases in 2017 was 4.5 times higher than that in 2000 (Fig. 2).

### Reasons for litigation

The main reasons for litigation were inappropriate clinical management in organization processes (46.1%), claimed misdiagnosis (22.6%), and subjective experienced poor nursing care (8.3%) (Table 2).

### Symptoms complained

The most common symptoms and complaints raised in the context of the litigation were mobility problems (37.2%), discomfort and pain (15.9%), and poor surgery outcome requiring reoperation (15.2%) (Table 3).

### Trends

The absolute number of litigations was *n* = 88 in the first time period (2000–2008) and *n* = 142 in the second time period (2009–2017). Compared with the first time period, there were significantly fewer litigations per 1000 cases in the second time period (56% less litigations; RR 0.44, CI 0.32–0.61, *p* < 0.0001).

### Elective surgery vs. trauma surgery

The average rate of litigations per 1000 cases was 1.09 in the first time period and 0.48 in the second time period for trauma surgeons. The average rate was 1.90 per 1000 cases for orthopedic surgeons in 2000–2008 and 0.83 after 2008. Significantly more litigations were filed against surgeons of the orthopedic subspecialties than against trauma surgeons (74% more litigations; RR 1.74, CI 1.26–2.42, *p* = 0.0001) (Table 4).

## Discussion

Our results could not confirm the often claimed increase in litigation procedures in the field of orthopedic and trauma surgery. Patients who underwent elective surgery were significantly more likely to file complaints than emergency patients. Well-designed studies about litigations in orthopedic and trauma surgery are rare. The present study aims to provide an overview of litigations using the example of a level I trauma center in Germany. Almost every surgeon will encounter lawsuits during his/her career. Jena et al. evaluated the risk of facing malpractice claims according to physician specialty. Surgical specialties always showed the highest probability of litigation. The risk of being sued as an orthopedic surgeon per year was almost twice as high (14%) as the average across all disciplines (7.4%) [17].

The most noticeable result of our study is the significant percentage decrease in litigations filed in our institution between 2000 and 2017. The absolute number of cases filed increased, potentially resulting in a subjective perception of a stark increase in litigation. However, the average rate of lawsuits per 1000 cases per year decreased from 1.54 in 2000–2008 to 0.67 in 2009–2017 because of an absolute increase in cases treated.

Many authors have described different results and increasing legal actions against surgeons. Reasons, such as poor quality of patient–physician relationship or the trend of considering medicine as a service that requires financial compensation, have been reported [2–5, 18]. Agout et al. found a significant increase in legal actions, from 9 cases in 2006 to 28 cases in 2015 (*p* = 0.04). Nevertheless, the authors presented absolute numbers of litigations per year only, and a possible change in the absolute number of treated cases was not reported. Erivan et al. reported an increase in patient complaints after arthroplasty between 2006 and 2016 from 0.2% up to 1.2% [5]. Cichost et al. analyzed a national legal database and found an increasing frequency of litigation (215%) and pay-outs (280%) during the period of 1988–2013 [18].

What are the potential reasons for the decrease in litigations in our collective? In 2012, our institution established a hospital customer service for both praise and complaint management. Studies have indicated that the introduction of a professional service for complaints is potentially important in patient satisfaction and quality of care [19, 20]. Eastaugh et al. stated that failed communication with patients and their families is the most common cause of malpractice suits [21].

Furthermore, many expert medical testimonies are performed in our institution, which is a university teaching hospital, thus giving it broad expertise in medical litigations. As all residents and senior surgeons are involved in the expert medical testimony procedures, the mental presence of potential malpractice procedures can lead to a higher level of alertness to avoid a lawsuit.

Finally, an international trend has emerged where the number of hospitals is being reduced in favor of fewer highly specialized centers. For most inpatient treatments, a higher volume was found to be associated with better patient safety and outcomes [22–25]. Erivan et al. showed an increase in the number of trauma surgeries and arthroplasties (43.6%) over a 10-year period, with no significant increase in complications [5]. The German Medical Association publishes the annual total figures of cases processed by the official arbitration board. The data showed stable values, with 10,705 claims in 2019, 12,053 claims in 2014, and 11,016 claims in 2010 [26]. Assuming an aging population with an increase in patient treatments per hospital, these data confirmed our results.

Another notable result is the significant difference in the number of litigations in trauma surgery compared with elective orthopedic surgery. After 2008, the estimated average rate of litigation was 0.48 per 1000 cases per year in trauma and emergency surgery and 0.83 in elective orthopedic surgery, such as spine surgery and total joint replacements (*p* = 0.0001). Tarantino et al. retrospectively analyzed 243 claims after orthopedic surgery and found a similar trend: elective surgery was responsible for 61% of litigations whereas only 39% of the claims were filed due to trauma surgery. Procedures most frequently involved in claims were total hip arthroplasty and lumbar decompression [27]. A study comparing a trauma department and 12 surgical specialties in a single center found the fewest events and lawsuits per 10,000 patients days for trauma patients. The authors conclude that despite the perception, trauma care has better claim experience than most surgical specialties [28]. A possible reason for the difference may be higher patient expectations prior to an elective surgery compared to an unforeseen operation due to an injury. Discrepancies between the patient and the surgeon regarding the expected result of a surgical procedure have often been described with consistently higher expectations on the patients’ side [29, 30].

The incidence of medical errors is difficult to determine because its definition differs depending on the study. Moreover, the frequency and type of errors vary greatly between the field of practice and the methods of detection in different publications [31]. The reported rate for complications in trauma surgery is 21.1%, with an error incidence of 8.7% [7]. Medically and legally, the line between a medical error and a hardly preventable complication can be very thin. Furthermore, the intention for legal action may depend on how serious the consequences of a medical error turned out to be, and therefore, not every case of medical malpractice will end in litigation or a malpractice claim. Stewart et al. evaluated the risk of malpractice lawsuits and compared elective, urgent, and trauma surgery. In contrast to our results, the authors found no significant difference between the groups and reported a low risk of litigation [32].

Finally, this study investigated the common reasons that trigger litigation. The main causes of litigation claims in elective and trauma surgery were inappropriate management/mistreatment at 46.5% for orthopedic patients and 45.2% for trauma patients and misdiagnosis/delay/failure to diagnose at 19.7% for orthopedic patients and 28.8% for trauma patients. Regarding the symptoms and complaints reported, mobility problems were the most common at 37.2%, followed by discomfort and pain at 15.9%. Other authors reported different values and reasons, such as surgical-site infection as the main reason at 50.7% [3] and failure in protecting structures in the surgical field [33, 34].

This study presents data from a German university hospital of orthopedic and trauma surgery and the litigations it faced over a period of 18 years. This work has several limitations. First, as each country has different laws for handling medical litigations, the results can only be generalized to a limited extent. Second, this is a retrospective single-center study, and no control group was included. For simplification, the reasons for litigation have been classified into categories. Thus, true reasons and motivations behind individual litigations can hardly be determined.

In conclusion, the findings could not confirm the often-stated trend of an increasing number of litigations against orthopedic and trauma surgeons. Claimed inappropriate management was the main reason for litigation in our institution. The risk of facing litigation was significantly higher for surgeons performing elective orthopedic surgery than for trauma surgeons.
